# Integrating physiological stress into the movement ecology of migratory ungulates: a spatial analysis with mule deer

**DOI:** 10.1093/conphys/coy054

**Published:** 2018-09-28

**Authors:** David S Jachowski, Matthew J Kauffman, Brett R Jesmer, Hall Sawyer, Joshua J Millspaugh

**Affiliations:** 1 Department of Forestry and Environmental Conservation, Clemson University, Clemson, SC, USA; 2 School of Life Sciences, University of KwaZulu-Natal, Westville, South Africa; 3 US Geological Survey, Wyoming Cooperative Fish and Wildlife Research Unit, Department of Zoology and Physiology, University of Wyoming, Laramie, WY, USA; 4 Program in Ecology, Department of Zoology and Physiology, University of Wyoming, Laramie, WY, USA; 5 Western Ecosystems Technology, Inc., 200 South 2nd St., Laramie, WY, USA; 6 W.A. Franke College of Forestry and Conservation, Wildlife Biology Program, University of Montana, Missoula, MT, USA

**Keywords:** Bottleneck, fecal glucocorticoid metabolites, fitness benefit hypothesis, green wave surfing, long-distance migration, movement ecology, stopover

## Abstract

Rapid climate and human land-use change may limit the ability of long-distance migratory herbivores to optimally track or ‘surf’ high-quality forage during spring green-up. Understanding how anthropogenic and environmental stressors influence migratory movements is of critical importance because of their potential to cause a mismatch between the timing of animal movements and the emergence of high-quality forage. We measured stress hormones (fecal glucocorticoid metabolites; FGMs) to test hypotheses about the effects of high-quality forage tracking, human land-use and use of stopover sites on the physiological state of individuals along a migratory route. We collected and analysed FGM concentrations from 399 mule deer (*Odocoileus hemionus*) samples obtained along a 241-km migratory route in western Wyoming, USA, during spring 2015 and 2016. In support of a fitness benefit hypothesis, individuals occupying areas closer to peak forage quality had decreased FGM levels. Specifically, for every 10-day interval closer to peak forage quality, we observed a 7% decrease in FGMs. Additionally, we observed support for both an additive anthropogenic stress hypothesis and a hypothesis that stopovers act as physiological refugia, wherein individuals sampled far from stopover sites exhibited 341% higher FGM levels if in areas of low landscape integrity compared to areas of high landscape integrity. Overall, our findings indicate that the physiological state of mule deer during migration is influenced by both anthropogenic disturbances and their ability to track high-quality forage. The availability of stopovers, however, modulates physiological responses to those stressors. Thus, our results support a recent call for the prioritization of stopover locations and connectivity between those locations in conservation planning for migratory large herbivores.

## Introduction

Animal migrations serve to bolster fitness by allowing animals to track shifting resource availability and predation risk, while balancing associated locomotive costs (Fryxell and Sinclair, 1988; Alerstam *et al.*, 2003). However, these behaviors are increasingly impacted by global change. For example, climate change has the potential to reduce the ability of large herbivores to accurately track or ‘surf’ gradients of forage quality (Post and Forchhammer, 2008). Trophic mismatch occurs when migratory species fail to optimally match peak resource availability with peak resource demands, often resulting in higher offspring mortality, lower parturition rates and lower population-level recruitment (Visser *et al.*, 1998; Post and Forchhammer, 2008; Rolandsen *et al.*, 2016). In addition, the ability of animals to accurately track forage quality can be negatively impacted by anthropogenic disturbance. For example, Rivrud *et al.* (2016) documented that the onset of human hunting activity was a stronger trigger for the start of migration by red deer (*Cervus elaphus*) than environmental cues (e.g. plant phenology, temperature, etc.). Similarly, disturbances along the migration route can negatively impact migratory movements, where animals move rapidly through or avoid areas of high human disturbance (Lendrum *et al.*, 2012; Sawyer *et al.*, 2013).

As migratory landscapes are altered by climate change, it is critical to develop conservation strategies that conserve or enhance the ability of individuals to track changing plant phenology (Sawyer and Kauffman, 2011; Merkle *et al.*, 2016). To best inform these conservation strategies, we increasingly require a mechanistic understanding of animal movement decisions (Wikelski and Cooke, 2006; Cooke *et al.*, 2008). The movement decisions of animals are governed by two primary processes, internal state and external environmental conditions (Nathan, 2008). However, traditional approaches to understanding the response of migratory large herbivores to landscape change focus primarily on assessing extrinsic conditions, such as the relationship between animal movement and anthropogenic disturbance or weather obtained via remote-sensing technologies (Jachowski and Singh, 2015). Further, studies limited to extrinsic influences typically only observe responses after the animal has already altered its migratory behavior or undergone population-level declines (e.g. Post and Forchhammer, 2008), meaning it may be too late for preemptive conservation action. Increasingly, it is understood that simultaneous investigations into both behavior and physiology are required to develop a more mechanistic understanding of how a continuum of fine-scale variations in migratory conditions influence subsequent movement decisions of migratory species (Jachowski and Singh, 2015).

Assessment of stress hormones (i.e. glucocorticoid metabolites) provides a tool to evaluate how multiple potential changes in extrinsic conditions influence the internal state of migratory species. While we are not aware of any previous attempts to monitor the stress physiology of long-distance migratory herbivores along their entire migration route, groups of large herbivores with elevated glucocorticoid stress hormones have been shown to exhibit altered movement patterns at both coarse (Jachowski *et al.*, 2012) and fine spatial scales (Jachowski *et al.*, 2013). Thus, assessing stress hormones in migratory large herbivores allows testing of important hypotheses about the complex, interactive effects of spatially and temporally changing landscape conditions on individuals along a migratory route.

While multiple factors can activate the hypothalamic–pituitary–adrenal axis and increase glucocorticoid (GC) production, we hypothesize that three primary spatially and temporally dynamic factors influence GC production for long-distance migratory herbivores (Table 1). First, GC production is known to be linked to energy balance in an individual, where GCs are released in response to negative energy balance (Kitaysky *et al.*, 2001; Wingfield and Kitaysky, 2002). Accordingly, we hypothesized that individuals would improve their energy balance, and thus lower their GC output as they progress along spring migration, particularly when foraging in habitats during phenological periods of peak forage quality (Hobbs, 1989; Fryxell, 1991; Parker *et al.*, 2005, 2009; Hamel *et al.*, 2009; Monteith *et al.*, 2011; 2013). Second, we hypothesized that high levels of human disturbance would result in a stress response and elevate GC concentrations in migratory herbivores (Millspaugh *et al.*, 2001; 2007; Creel *et al.*, 2002; Jachowski *et al.*, 2012; 2013). Third, mule deer use stopovers to stay in pace with peak forage quality during migration (Sawyer and Kauffman, 2011), which could improve energy balance and result in decreased circulating GC concentrations. Accordingly, we hypothesized that stopovers act as physiological refugia along the migration route, allowing long-distance migratory herbivores to enhance tracking of peak forage quality and reduce GC production.

**Table 1:** Tested *a priori* hypotheses and predictions of the relationship between environmental and anthropogenic factors and observed fecal glucocorticoid metabolite (FGM) concentrations in mule deer sampled during spring migration between the Red Desert and Hoback Basin in Wyoming. Landscape integrity (LI) was scaled from 0 to 1, where high values indicate low human disturbance (Copeland *et al.* 2007). Instantaneous rate of green-up (IRG) is a proxy for forage quality, where previous research has suggested temperate ungulates should select for a specific point on the migration route closest to the date of peak IRG (Bischof *et al.* 2012; Merkle *et al.* 2016). Stopovers were identified based on long-term movement data (Sawyer *et al.* 2014; Fig. 1), negative values for stopover site distance indicate locations within the stopover area boundary or edge, values at 0 represent locations exactly on the boundary or edge of a stopover, and positive values indicate distances outside of a stopover area

Hypothesis	Prediction	Metrics used (predicted relationship with GC)	Citations
*Fitness benefit hypothesis*	Individuals that track peak forage quality well would improve their energy balance and reduce GC output.	Distance from start (−), NDVI (−), IRG (−), Days to peak IRG (+)	Hobbs (1989), Parker *et al.* (2005, 2009), Monteith *et al.* (2011, 2013)
*Additive anthropogenic disturbance hypothesis*	Individuals exposed to high levels of human disturbance would exhibit higher GC concentrations.	Landscape integrity (−)	Millspaugh *et al.* (2001, 2007), Creel *et al.* (2002), Jachowski *et al.* (2012, 2013)
*Physiological refugia hypothesis*	Stopover utilization had a negative influence on GC production in long-distance migratory herbivores	Distance from center of stopover (+)	Sawyer and Kauffman (2011)

To test these hypotheses, we assessed non-invasive fecal glucocorticoid metabolite (FGM) data from a migratory mule deer population in western Wyoming, USA. This population is known to exhibit stopover behavior as well as to navigate several anthropogenic and naturally occurring bottlenecks with varying levels of human disturbance (Sawyer *et al.*, 2014). Further, studies of this population and those nearby suggest that mule deer in this region coordinate their movements with the phenological progression of plants in an attempt to track forage quality (Merkle *et al.*, 2016; Aikens *et al.*, 2017), make use of stopover sites, and alter their movement in response to intensive anthropogenic disturbance (Sawyer and Kauffman, 2011; Sawyer *et al.*, 2013, 2014; Wyckoff *et al.*, 2018). This population therefore represents an ideal system to evaluate support for hypotheses about the physiological response of migratory large herbivores to tracking high-quality forage in a human-modified landscape.

## Materials and Methods

### Study site

**Figure 1:**
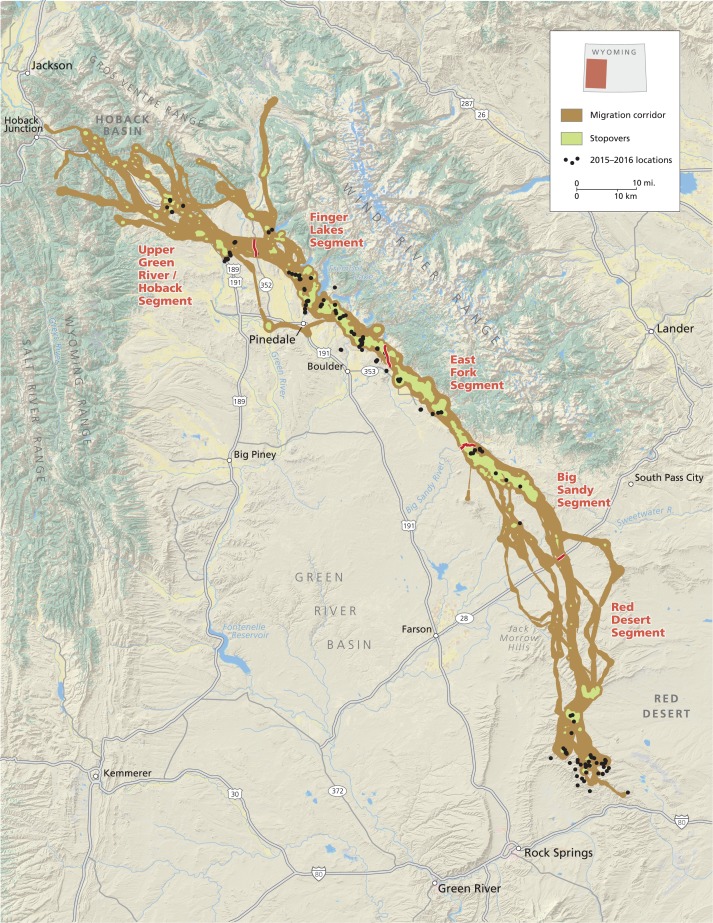
Red Desert to Hoback Basin mule deer migration route (brown) and stopover sites (green), Wyoming. Black points indicate locations where fecal samples were collected in March and April, 2015 and 2016. The 241-km migratory route can be classified into five segments, labeled in red. The inset figure contains the location of the study area within the State of Wyoming, USA.

We focused our study on the 241-km South-to-North spring migration of mule deer that begins in the Red Desert where an estimated 1500 mule deer overwinter near Rock Springs, Wyoming (Fig. 1). Mule deer overwinter in this area due to its low elevation (~900–1200 m difference from summer range), with relatively moderate temperatures and less snowfall during winter compared to the northern portion of their migratory route (see Sawyer *et al.* (2014) for a more detailed description of migratory route conditions). Beginning in March–June each year (depending on weather conditions and distance to summer range) individuals travel 80 km North across the Red Desert to the base of the Wind River Mountain Range (Fig. 1). At that point, deer often intermix with an additional 4000–5000 migratory mule deer that overwinter at the southern end of the Wind River Range. From there, migrating mule deer continue along the western edge of the Wind River Range for 154 km into the Green River Valley, crossing the Green River Basin, prior to climbing up to their summer range in the Hoback Basin (Sawyer *et al.*, 2014; Fig. 1).

The migratory route can broadly be described in five key segments based on differences in environmental conditions and anthropogenic disturbance (Fig. 1; Sawyer *et al.*, 2014). Going from South to North, the Red Desert region is primarily composed of federally-managed rangelands (70%) with only one fence crossing and minimal human development or disturbance (Sawyer *et al.*, 2014). The Big Sandy segment is distinct, based on the transition from lower topography of the Red Desert to rolling sagebrush-dominated (*Artemisia* sp.) hills and basins that lead up to the base of the Wind River Range. The approximately 40-km long section begins with the crossing of Highway 28 (two-lane highway with average daily traffic volume (ADTV) of 1 238 in 2016 (WYDOT 2016)) and is primarily composed of federally-managed rangelands (90%) with little human disturbance, but contains eight fence crossings (Sawyer *et al.*, 2014). The East Fork segment, also ~40 km long, begins North of the Big Sandy River and is composed of sagebrush foothills of the Wind River Range that contain extensive known stopover locations. This segment has diverse land ownership (43% private, 38% public and 19% state lands) managed primarily for livestock grazing and energy development (Sawyer *et al.*, 2014). The Finger Lakes segment is distinct, characterized by natural lakes that protrude down into the migration route from the mountains, forcing deer into a series of bottlenecks 50–400 m wide (Fig. 1). Land ownership is 44% federal, 46% private (of which 9% has been placed under conservation easement) and 8% state-managed land (including several wildlife management areas; Sawyer *et al.*, 2014). The Fremont Lake bottleneck in this section is particularly noteworthy because the town of Pinedale at the foot of the lake likely represents the area of highest human disturbance along the migratory route. Deer must cross several roads in this section, including Highway 352 (two-lane highway with ADTV of 525 in 2016 (WYDOT 2016)), prior to entering the Upper Green River-Hoback segment (Fig. 1). This final 50–75 km segment is primarily (52%) composed of privately managed rangelands in the Green River Valley (Sawyer *et al.*, 2014). Deer typically cross the Green River as well as a major highway, US Route 189/191 (two-lane highway with ADTV of 2 238 in 2016 (WYDOT 2016)), to reach federally-managed forest lands in the North and northwest where they reside for the summer.

### Field sampling

We conducted field surveys and collected fresh fecal samples for stress hormone analysis during the spring (northward) migration in 2015 (14–27 April ) and 2016 (16–24 March). Given that mule deer along this migratory route exhibit a range of migratory strategies (i.e. starting times, starting points and movement speed), we selected these periods during the peak of migratory activity in an effort to collect samples over a range of times and locations from deer along the entire Red Desert to Hoback (RDH) migratory route. To collect fresh samples (<24 h old), we systematically traveled portions of the migratory route each day and opportunistically observed mule deer. We never tracked the same portion of the route on consecutive days and thus avoided sampling deer we might have unintentionally disturbed. Upon encountering deer (all individuals were adults or juveniles), we watched them at a distance >100 m until they moved out of view; then walked the area in ~5 m transects to locate and collect fresh fecal samples. Only samples <24 h old were collected based on seeing the defecation occur, or on the appearance and texture of the pellets. Samples were almost always clustered in discrete piles, and we attempted to only collect one sample from each individual based on a combination of pile location, sample age and/or bolus size. Samples were labeled, stored on ice in the field and within 8 h placed in a −80°C freezer prior to shipment to the laboratory.

### Laboratory analysis

We placed frozen fecal samples in a lyophilizer (Freeze-dry Specialties, Inc. Osseo, Minnesota, USA) for 24 h. Freeze-dried samples were sifted through a stainless steel mesh to remove large debris, with ~0.20 g placed in a test tube with 2.0 ml of 90% methanol and vortexed at high speed in a multi-tube vortexer for 30 min. Samples were centrifuged at ~1900 *g* for 20 min, and the supernatant stored at −84°C until assayed.

We used I125 corticosterone radioimmunoassay (RIA) kits (ICN #07–120 103, ICN Biomedicals, Costa Mesa, CA, USA) previously validated for use with white-tailed deer (Millspaugh *et al.*, 2002) to quantify FGM concentrations. Based on experimental evaluations of captive cervids, FGMs likely represent long-term physiological state and the response to acute stressor at least 8–30 h prior (Wasser *et al.*, 2000; Dehnhard *et al.*, 2001; Millspaugh *et al.*, 2002). We followed the ICN protocol for the I125 corticosterone RIA, except that we halved the volume of all reagents (Wasser *et al.*, 2000).

We conducted a standard assay validation including assessment of parallelism, recovery of exogenous analyte, intra- and inter-assay precision and assay sensitivity (Jeffcoate, 1981; Grotjan and Keel, 1996; O’Fegan, 2000) to confirm that the assay accurately and precisely measured corticosterone metabolites in mule deer feces. Parallelism and recovery of exogenous corticosterone validation assays were conducted on two pooled fecal extract samples (each pool consisted of feces from five individuals). Parallelism ensures the assay maintains linearity under dilution, and recovery of exogenous corticosterone verifies accurate measurement throughout the working range of the assay (Jeffcoate, 1981). We added exogenous corticosterone to the low and high pooled fecal extracts to obtain corticosterone values under higher dilution levels (each pool consisted of feces from five individuals). We used tests for equal slopes (parallelism) to determine if log-transformed curves of serially diluted pooled fecal extracts were parallel to log-transformed corticosterone standard curves. We used the low and high controls provided with the kits and analysed them in all assays. Inter-assay variation was calculated from these two controls by averaging the coefficient of variation of replicate tubes from 20 randomly chosen samples.

Assay sensitivity was 1.25 ng/g. The manufacturer’s reported cross-reactivity of the antisera was 100% with corticosterone and <1% for other steroids. For fecal samples collected in 2015 (*n* = 229), inter-assay variation was 6.7% and average intra-assay variation was 1.4%. For fecal samples collected and analysed in 2016 (*n* = 170), inter-assay variation was 6.3% and average intra-assay variation was 1.0%.

### Hypothesis testing

We developed a set of *a priori* candidate models used to predict GC values based on the phenological stage of the habitat occupied by sampled deer, anthropogenic disturbance and the use of stopovers along the migratory route (Table 1).

#### Fitness benefit hypothesis

For most migratory herbivores occurring in temperate biomes, winter is known to be the period of extreme negative energy balance, and spring migration is undertaken at the beginning of the growing season when energy balance turns positive (Parker *et al.*, 2009). Additionally, forage quality and energy balance are predicted to increase if an animal tracks peak forage quality from winter range to summer range during migration (Hobbs, 1989; Parker *et al.*, 2005, 2009; Hamel *et al.*, 2009; Monteith *et al.*, 2011). Therefore, we first tested support for the Fitness Benefit Hypothesis which suggests that individuals improve their energy balance and thus lower their GC output as they progress along spring migration and forage in habitats that are near peak forage quality.

We first predicted that animals would experience greatest energy deficits at the end of winter while still on winter range (Hobbs, 1989; Parker *et al.*, 2005). While we were unable to identify individual deer and were therefore unable to know the individual’s specific migration strategy or winter range, we predicted that those individuals toward the southern end of the migration route would be closest to their winter range because the migration of study population progresses from South to North (Fig. 1). Therefore, deer in the southern part of the migration corridor should exhibit greater energy deficits and have the highest GC concentrations.

Second, long-distance migratory herbivores also make fine-scale movement decisions along their migration route in an effort to track peak forage quality (Merkle *et al.*, 2016; Aikens *et al.*, 2017). Therefore, we included an effect of two fine-scale metrics of forage availability and quality along the migratory route. We used rasterized satellite imagery (surface reflectance Bands 1 and 2 from MODIS product MOD09Q1; 250 m spatial and 8-day temporal resolution) to calculate the normalized difference vegetation index (NDVI). We interpreted NDVI as an index of greenness scaled between 0 and 1 that correlates with standing plant biomass (Pettorelli *et al.*, 2005). To calculate NDVI for each location and time interval of interest, we followed methods summarized in Merkle *et al.* (2016), where within each pixel we calculated a time series of changing plant phenology where 1 represented peak greenness. Using this time series of NDVI raster grids, we computed the date at which forage reached peak forage quality by calculating the Instantaneous Rate of Green-up (IRG; Bischof *et al.* 2013).

For mule deer and several other large herbivores in our study region, IRG has been shown to be a better predictor of migratory movement decisions by herbivores than NDVI (Merkle *et al.*, 2016). IRG provides an index of forage quality and builds on the increasing evidence for the forage maturation hypothesis, which states that herbivores should select for forage at an intermediate state of phenological growth when plant digestibility and nutrient content are highest to optimize nutritional gain (Fryxell, 1991; Bischof *et al.*, 2012). Effectively, NDVI values peak at 1 when total biomass is at a maximum, while IRG is the first derivative of the NDVI fitted annual curve and better approximates the point at which a given pixel is at intermediate green-up and likely to have highest quality forage (Bischof *et al.*, 2012; Merkle *et al.*, 2016). Accordingly, we tested the prediction that animals occupying habitats when they were close to peak IRG would exhibit lower GC levels. In addition, given the transient nature of animals along the migratory route, we were interested in the number of days away from peak IRG that a location was used (Aikens *et al.*, 2017). Specifically, we tested the prediction that animals better able to occupy a specific point on the migration route closest to the date of peak IRG at that location would exhibit lower GC levels compared to animals that arrived prior to or after the peak IRG date.

Finally, we predicted that the fitness benefit hypothesis could influence GC production at both large and fine spatial scales—where the relative influence of IRG tracking ability on GC would be greatest during early periods of migration as mule deer left winter range and likely were in their poorest negative energy balance. Therefore, we evaluated support for an interaction between maximum migration distance from winter range (i.e. distance from the farthest south winter range for migrants on the RDH) and days to peak IRG.

#### Additive anthropogenic disturbance hypothesis

Glucocorticoid production is known to oscillate within a range of values in response to a suite of intrinsic (e.g. energy balance) and extrinsic (e.g. weather, anthropogenic disturbance) stressors, yet stochastic events or the introduction of novel stressors can lead to elevated GC levels (Wingfield *et al.*, 1997; McEwen and Wingfield, 2003; Sheriff *et al.*, 2009; Boonstra, 2013). In large herbivores, human disturbance has been demonstrated to act as a stressor and elicit a short-term and longer-term elevation in stress hormone production (Millspaugh *et al.*, 2001, 2007; Creel *et al.*, 2002; Jachowski *et al.*, 2012, 2013). Similarly, we hypothesized that high levels of human disturbance would elevate GC concentrations in migratory herbivores. We evaluated support for a hypothesized additive effect of human disturbance on GCs by using an index of landscape integrity that integrated measures of urban development, agriculture, roads, and energy development (Table 1). This single weighted index was developed by Copeland *et al.* (2007; and updated in 2016) at the 30 m grid scale, where a value of 1 indicates an undisturbed location, and 0 indicates highly disturbed. We also hypothesized that the influence of poor tracking of high-quality forage could interact with anthropogenic disturbance, where we predicted that animals in areas of low landscape integrity and occupying such habitats much earlier than peak forage quality would exhibit heightened GC levels.

#### Physiological refugia hypothesis

The physiological response of individuals that poorly track high-quality forage and experience anthropogenic disturbance is likely modulated or exacerbated by stopover behavior during migration. For example, in response to high levels of human disturbance, African elephants (*Loxodonta africana*) exhibit elevated GC concentrations and rapidly move through or otherwise avoid areas associated with human disturbance in favor of forested areas that act as refugia (Jachowski *et al.* 2013). There is some evidence that migratory ungulates exhibit similar behaviors. For example, pronghorn (*Antilocapra americana*) delay progress along their migration route just ahead of anthropogenic barriers (e.g. fences) or bottlenecks (Seidler *et al.*, 2015), suggesting that use of stopover behavior could be associated with perceived disturbance and elevated GC concentrations. By contrast, mule deer use stopovers to stay in pace with peak forage quality during migration (Sawyer and Kauffman, 2011), which could improve energy balance and result in decreased circulating GC concentrations.

To evaluate support for our physiological refugia hypothesis, where stopover utilization was predicted to improve energy balance and reduce GC production in long-distance migratory herbivores, we measured the distance a sample was collected from the center of a stopover site based on multiple years of GPS-tracking data (Sawyer *et al.*, 2014). In addition, because human disturbance varied along the migration route, we evaluated support for an interaction between stopover distance and landscape integrity, where under the physiological refugia hypothesis we would predict that deer farther away from stopovers in areas of low landscape integrity would exhibit higher GCs than deer using stopovers in areas of high landscape integrity. We also predicted that under the physiological refugia hypothesis, stopovers could mitigate the physiological stress response an individual mounts in response to occupying those sites prior to peak forage quality. Therefore, we evaluated support for an interaction between stopover distance and days to peak IRG, where we predicted that samples collected from animals that occupied sites either before or after peak IRG and were located farther from stopovers would exhibit higher GC concentrations than those who similarly mistimed arrival but were within or near stopover sites.

### Model fitting

We used general linear mixed-effects models in an information theoretic model selection framework to evaluate support for 31 competing *a priori* models (Burnham and Anderson, 2002). We treated FGM values from each sample collected as the response variable, and included a random effect for group that was sampled. We used the term ‘group’ loosely here, as the total number of individuals was not always visible and samples were not collected from all animals observed. Rather, group refers to a cluster of samples collected at a site from different individuals (based on bolus size, proximity and age of sample) following the observation of one or more animals at the same place at the same time. We considered all model(s) that contributed to 90% of cumulative model weight as having evidence of support. For our top-ranked models, we calculated the variance explained by fixed effects only (marginal *R*^2^) and fixed and random effects (conditional *R*^2^) using the ‘MuMIn’ package in Program R (Barton, 2018).

## Results

We collected and analysed 399 samples from 78 mule deer groups along the migration route between 14–27 April 2015 and 16–24 March 2016 (Fig. 1). We collected samples from across the migration route (Fig. 1), with 53 samples collected in Segment 1 (mean = 66.7, SD = 15.2), 30 in Segment 2 (mean = 68.8, SD = 18.5), 60 in Segment 3 (mean = 54.5, SD = 17.7), 194 in Segment 4 (mean = 55.5, SD = 19.0) and 62 in Segment 5 (mean = 63.3, SD = 16.7).

We observed support for two models in our confidence set, but the top model contained three times as much support as the next most supported model, and was more parsimonious than the second supported model which only added a quadratic effect of dIRG (Table 2). Thus, we focused our interpretation using parameters retained in our top model, which included an effect for days to maximum or peak IRG and an interaction between landscape integrity and distance to nearest stopover (Table 3).

**Table 2:** Support for models used to predict fecal glucocorticoid metabolite concentrations in mule deer sampled during migration between the Red Desert and Hoback Basin in Wyoming in April 2015 and March 2016. Only the two models that contributed up to 90% of cumulative model weight are reported. For our top-ranked models, we calculated the variance explained by fixed effects only (marginal *R*^2^) and fixed and random effects (conditional *R*^2^)

Model structure	K	AIC_c_	ΔAIC_c_	Model likelihood	AIC_c_ weight	Log likelihood	Marginal *R*^2^	Conditional *R*^2^
= β_1_(dIRG) + β_2_(LI) + β_3_(STPOVR) + β_4_(LI * STPOVR)	7	42.36	0.00	1.0000	0.6767	−14.04	0.1568	0.4786
= β_1_(dIRG) + β_2_(dIRG^2^) + β_3_(LI) + β_4_(STPOVR) + β_5_(LI * STPOVR)	8	44.42	2.06	0.3569	0.2415	−14.03	0.1564	0.4782

**Table 3:** Parameter coefficients (and standard error) from top-ranked models used to predict fecal glucocorticoid metabolite concentrations in mule deer. Models and parameter coefficient notation is explained in Tables 1 and 2

	Model 1	Model 2
Intercept	4.0547 (0.0377)	4.0516 (0.0430)
dIRG	−0.1104 (0.0229)	−0.1089 (0.0249)
dIRG^2^		0.0029 (0.0194)
LI	−0.0780 (0.0553)	−0.0779 (0.0553)
STPOVR	0.0824 (0.0281)	0.0822 (0.0282)
LI*STPOVR	−0.1801 (0.0590)	−0.1790 (0.0595)

**Figure 2:**
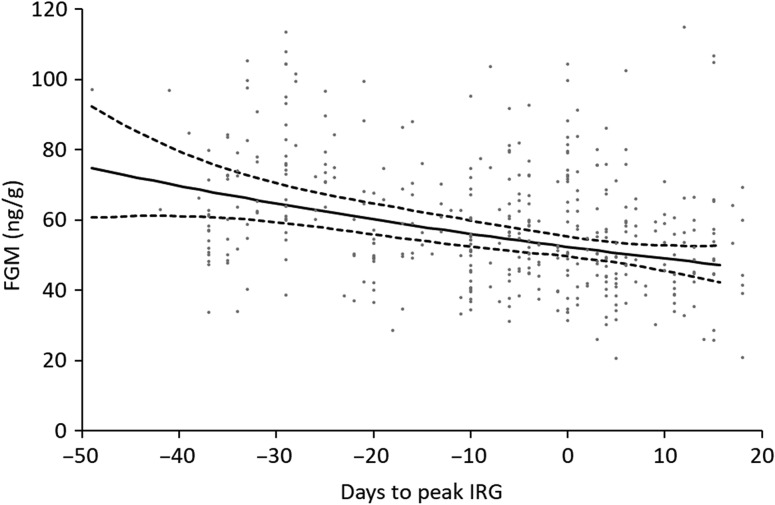
Estimated relationship (with dashed lines representing 95% confidence intervals) between fecal glucocorticoid metabolite (FGM) concentrations and when a mule deer used a location in relation to the peak or maximum instantaneous rate of green-up (IRG) value at that site along the northward migration between the Red Desert and Hoback Basin in Wyoming, USA. IRG is a proxy for forage quality (Bischof *et al.* 2012; Merkle *et al.* 2016), where peak IRG is indicated by day 0 and was determined for each location along the migration route where a sample was collected. Negative values indicate days prior to peak IRG, and positive values indicate days after peak IRG was observed at a site. Estimated relationship is based on top predictive model with all other covariates held at their mean and points in background represent observed FGM values (*n* = 399).

In support of our fitness benefit hypothesis, FGM production was estimated to be lower for individuals occupying habitats at peak or after peak forage quality when high-quality forage was available (Fig. 2). For every 10 days prior to peak IRG that a mule deer sample was collected at a site, we observed a 7% increase on average in FGM levels (Fig. 2).

**Figure 3:**
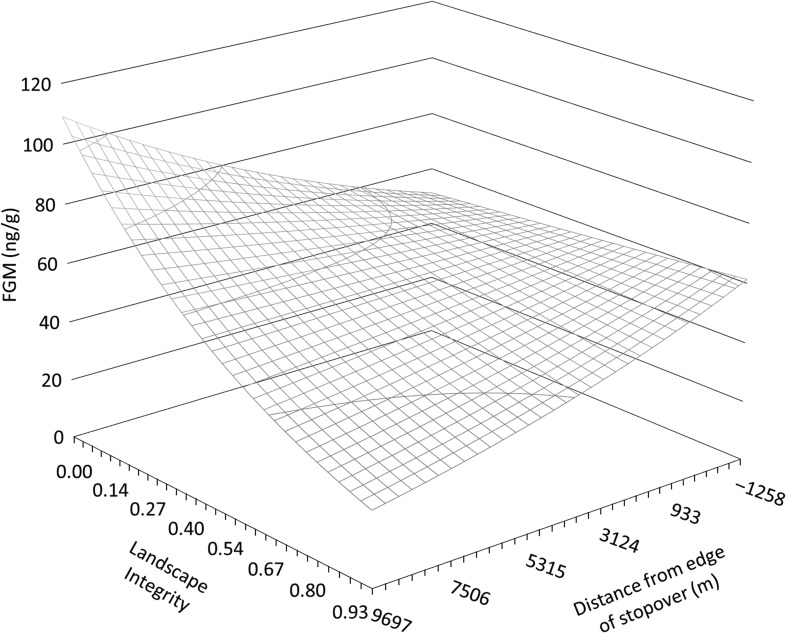
Estimated relationship between the interactive effect of landscape integrity and distance from the edge of a stopover site on migratory mule deer fecal glucocorticoid metabolite (FGM) levels. The estimated relationship is based on top predictive model with all other covariates held at their mean. Stopovers were identified based on long-term movement data (Sawyer *et al.* 2014; Fig. 1), negative values for stopover site distance indicate locations within the stopover area boundary or edge, values at 0 represent locations exactly on the boundary or edge of a stopover, and positive values indicate distances outside of a stopover area. Landscape integrity is scaled from 0 to 1, where high values indicate low human disturbance (Copeland *et al.* 2007).

**Figure 4:**
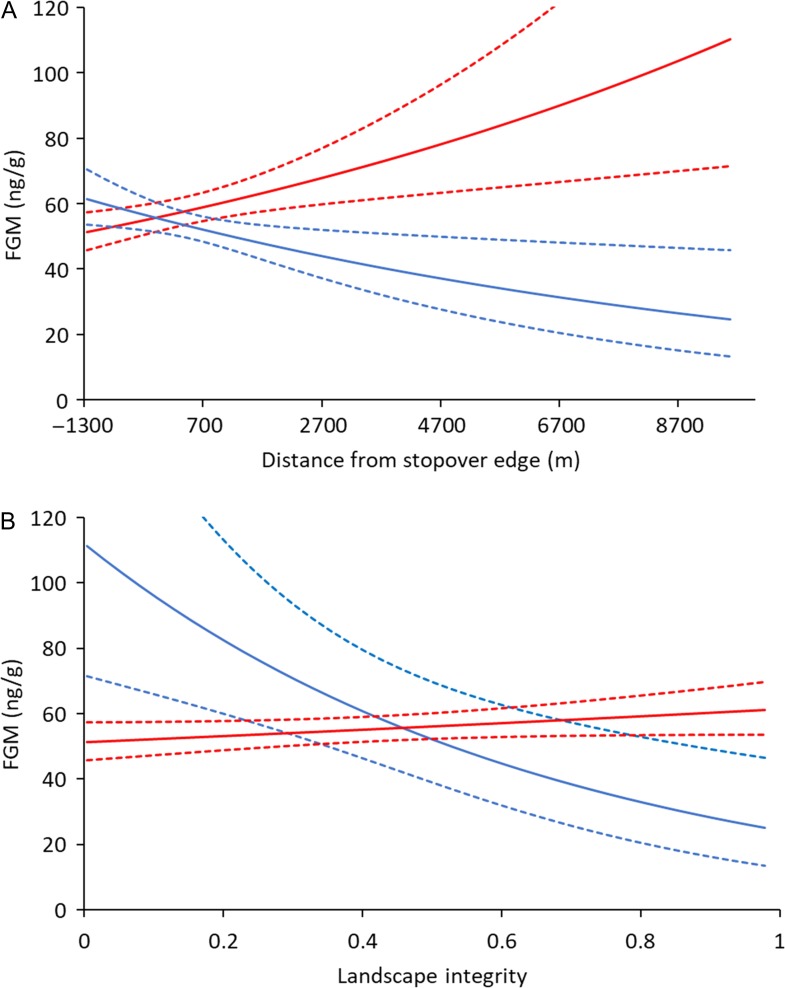
Estimated relationship (with dashed lines representing 95% confidence intervals) between the interactive effect of distance from the edge of a stopover site and landscape integrity on migratory mule deer fecal glucocorticoid metabolite (FGM) levels. The estimated relationship is based on top predictive model with all other covariates held at their mean. The upper figure (**A**) displays predicted FGM values as a function of landscape integrity (0 represents high human disturbance, 1 represents areas with no human disturbance) when distance from stopover is held at its minimum (red) and maximum (blue). Dashed lines represent 95% confidence intervals. The lower figure (**B**) displays predicted FGM values as a function of distance for stopover edge (negative values indicate locations further within the edge of stopovers) when landscape integrity is held at its minimum (red) and maximum (blue).

In support for our additive anthropogenic stress and physiological refugia hypotheses, we observed a dampening effect of stopover use on FGMs, particularly when individuals were sampled in areas of high human disturbance (Fig. 3). We observed that animals sampled far from stopovers on average were predicted to have 341% higher FGMs in areas of low landscape integrity compared to high landscape integrity (Fig. 4a). By contrast, we observed that for samples collected from within stopovers, FGMs were 20% lower in areas of low compared to high landscape integrity (Fig. 4a). Focusing exclusively on areas of low landscape integrity, FGMs in samples collected 7000 m from the edge of a stopover were, on average, estimated to be 80% higher compared to samples collected 1200 m within the edge of a stopover (Fig. 4b). By contrast, in areas of high landscape integrity over a similar range of stopover distance, FGMs were, on average, estimated to be 101% higher in stopover areas (but still below 60 ng/g; Fig. 4b). Overall, our findings suggest that stopover use in this system served as physiological refugia along the migration route in areas of high human disturbance.

## Discussion

We observed empirical support for our fitness benefit hypothesis which indicates that accurate tracking of high-quality forage along a migration route has a physiological benefit for migratory herbivores. The pattern we observed of FGMs being elevated for individuals using areas well ahead of peak forage quality supports previous predictions that energy balance is enhanced by tracking peak forage quality during migration from winter to summer range (Hobbs, 1989; Parker *et al.*, 2005, 2009; Monteith *et al.*, 2011; 2013). However, the act of migration (i.e. distance from southernmost winter range) itself was not a strong predictor of GC production, suggesting that the timing of when an individual starts a migration or total amount of progress along the route is less critical than the finer-scale movement decisions made along the migratory route aimed at tracking high-quality forage. Thus, when considered in concert with previous work that illustrates the generality of optimal spatial and temporal tracking of peak forage quality by migratory herbivores (Merkle *et al.*, 2016; Aikens *et al.*, 2017), there is increasing evidence that an individual’s ability to track or ‘surf’ high-quality forage during spring green-up likely has broad fitness benefits.

Our assessment of stress hormones provided insight into stopover behavior, and the critical role stopovers likely have in mitigating stress responses resulting from anthropogenic disturbance. Evidence of elevated FGMs in samples collected in areas of high human disturbance, but away from stopover sites, offers support for the physiological refugia hypothesis and leads to one or both of the following conclusions. First, it could provide further evidence that stopovers are critical to the timing of tracking high-quality forage and to the maximizing of energy and nutrient intake along a migratory route (Sawyer and Kauffman, 2011). Second, and in contrast to previous hypotheses suggesting that stopovers are primarily associated with the constriction of migratory corridors by human land-use change (Seidler *et al.*, 2015), we observed that stopovers play a key role in dampening the additive effect of human disturbance on the physiological stress response of migratory species. We note, however, that differences in stopover use between pronghorn (see Seidler *et al.*, 2015) and mule deer (this study) may also be influenced by species-specific movement constraints (i.e. mule deer can jump over fences, whereas pronghorn do not). Nonetheless, our findings support previous research that calls for conservation planning that prioritizes stopover locations and maintenance of connectivity between those locations (Sawyer and Kauffman, 2011; Aikens *et al.*, 2017), particularly in areas of high anthropogenic disturbance.

Within stopovers, we found that FGMs were slightly elevated in landscapes with high landscape integrity (i.e. low human activity), which was surprising because anthropogenic disturbance has been demonstrated to influence the stress axis of a wide variety of vertebrate taxa (Busch and Hayward, 2009), including large herbivores (Millspaugh *et al.*, 2001; 2007; Creel *et al.*, 2002; Jachowski *et al.*, 2012; 2013). The lack of support for this prediction was likely a result of (1) stopovers near human disturbance in our study area having limited human presence due to management regulations and (2) our inability to directly link FGM levels with the energetic state of individuals or their fine-scale movement, foraging and migratory strategies. While individual state is not thought to influence when an individual mule deer initiates spring migration (Monteith *et al.*, 2011) or its ability to track high-quality forage (Aikens *et al.*, 2017), state-dependent responses to elevations in GC concentrations could be occurring at finer spatial scales (Jesmer *et al.*, 2017). Thus, one explanation could be that mule deer were foraging in a state-dependent manner, wherein individuals with negative energy-balance that just arrived at a stopover site exhibited elevated GCs. Further, elevations in GC have been attributed to increased foraging activity or altered diet selection (Kitaysky *et al.*, 1999; 2001; Jesmer *et al.*, 2017). Thus, acute stress responses could be playing a mechanistic role in decisions by animals to remain at stopovers on the basis of nutritional demands, which is in line with our current understanding of the role of GCs in energetic balance (i.e. allostasis; McEwen and Wingfield, 2003; Busch and Hayward, 2009).

Overall, given the rapidly increasing rate of land-use change along corridors for many long-distance migratory species across the globe (Berger, 2004), there is a clear need to understand how the additive effect of anthropogenic disturbance on GC could influence the physiology and behavior of migratory animals. Chronic activation of the HPA axis and elevated GC concentrations can result in a suite of fitness consequences including decreased survival and recruitment (Boonstra *et al.*, 1998; Blas *et al.*, 2007; Sheriff *et al.*, 2009). Thus, on the continuum of potential responses by an animal to a stressor, GC is likely the most sensitive indicator of potentially detrimental consequences of a stressor (Creel *et al.*, 2002). A key next step in the conservation of migratory species is to assess if individuals exhibit long-term elevations in GC production, and if there is a threshold beyond which chronic elevations in GC production results in decreased fitness and altered demography. Further, this population and other migratory herbivores are known to exhibit a variety of migratory strategies ranging from long-distance to partial or short-distance migrations (Cagnacci *et al.*, 2011; Chapman *et al.*, 2011; Sawyer *et al.*, 2016). Thus, a logical next step is to evaluate if there are physiological thresholds at which point migratory individuals switch their migratory behavior from long- to short-distance or to year-round residency in response to land use and climate change.

In conclusion, our study of FGMs in a long-distance migratory herbivore allowed us to gain several important, novel insights into the response of individuals to human activity along their migration corridor. This initial correlative study provides support for further, direct experimental testing of the mechanistic role GC might play in animal decision making during migration. For example, a key next step is to use GPS-tracking to link individual behavior to physiology throughout a migration and across differing migration strategies and seasons. Regardless, our findings suggest that during spring migration, populations appear to be responding to forage quality and anthropogenic disturbance, both individually and interactively, and that the availability of stopovers likely reduces physiological stress. Further, our findings suggest that monitoring of FGM concentrations along a migration route can be used not only to target areas where conservation action might be needed for migratory herbivores, but also to evaluate the success of conservation actions. More generally, given the threats that animal migrations face across the globe, our work highlights the potential for future pairing of movement and remote-sensing data with non-invasive physiological monitoring to help better understand migratory behavior and to guide future conservation efforts.
